# Combined behavioral and EEG evidence for the 70 Hz frequency selection of short‐term spinal cord stimulation in disorders of consciousness

**DOI:** 10.1111/cns.14388

**Published:** 2023-08-11

**Authors:** Yutong Zhuang, Qianqian Ge, Qinghua Li, Long Xu, Xiaoli Geng, Ruoqing Wang, Jianghong He

**Affiliations:** ^1^ Department of Neurosurgery Beijing Tiantan Hospital, Capital Medical University Beijing China; ^2^ Department of Neurosurgery The Second Clinical College of Southern Medical University Guangzhou China; ^3^ College of Anesthesiology Shanxi Medical University Taiyuan China; ^4^ High School Affiliated to Renmin University Beijing China

**Keywords:** disorders of consciousness, short‐term spinal cord stimulation, frequency, EEG, functional connectivity, brain network

## Abstract

**Objectives:**

This study investigated the prognostic effect of electroencephalography (EEG) instant effects of single spinal cord stimulation (SCS) on clinical outcome in disorders of consciousness (DOC) and the time‐dependent brain response during the recovery of consciousness prompted by SCS.

**Methods:**

Twenty three patients with DOC underwent short‐term SCS (stSCS) implantation operation. Then, all patients received the postoperative EEG test including EEG record before (T1) and after (T2) single SCS session. Subsequently, 2 weeks stSCS treatment was performed and revised coma recovery scale (CRS‐R) and EEG data were collected. Finally, they were classified into effective and ineffective groups at 3‐month follow‐up (T6).

**Results:**

The parietal‐occipital (PO) connectivity and clustering coefficients (CC) in the beta band of the effective group at the 1 week after the treatment (T5) were found to be higher than preoperative assessment (T0). Correlation analysis showed that the change in beta CC at T1/T2 was correlated with the change in CRS‐R at T0/T6. In addition, the change in PO connectivity and CC in the beta at T0/T5 were also correlated with the change in CRS‐R at T0/T5.

**Conclusion:**

SCS may facilitate the recovery of consciousness by enhancing local information interaction in posterior brain regions. And the recovery can be predicted by beta CC in the EEG test.

## Graphical abstract

We combined dynamic changes of EEG induced by a single SCS session with behavioral evidence to explore the immediate feedback indicators for frequency selection. The change in beta CC before and after single SCS session can predict the long‐term clinical outcome of 70 Hz stSCS. SCS may facilitate the recovery of consciousness by enhancing local information interaction in posterior brain regions. EEG, electroencephalography; SCS, spinal cord stimulation; stSCS, short‐term SCS; CC, clustering coefficients.

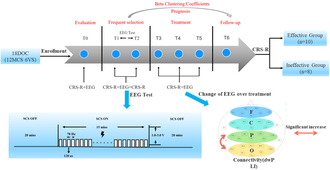

## 1 INTRODUCTION

Disorders of consciousness (DOC) are caused by various brain injuries that result in the loss of an individual's ability to perceive surroundings and his/her own state. DOC can be divided into two dimensions of arousal and content of consciousness. The vegetative state (VS) is characterized by an abnormal sleep–wake cycle, whereas the autonomous consciousness is imperceptible. The minimally consciousness state (MCS) further recovers from VS with repeatable but fluctuating signs of consciousness, and is subdivided into two levels of consciousness: MCS– and MCS+.^1^ A previous positron emission tomography study confirmed that MCS+ patients retained more metabolism in the right brain areas related to language and communication functions compared with MCS– patients.^2^

Treatment of DOC has been a major bottleneck due to permanent necrosis of large thalamic and cortical neurons and extensive disconnection of long‐range connections of the thalamocortical network.^3^, ^4^ In this regard, SCS is expected to be a key therapeutic measure to achieve stable improvement in consciousness owing to its ability to directly modulate the thalamic–cortical neural circuit. A 2009 large‐sample study reported that 54% of VS patients had a good prognosis after long‐term treatment with 70 Hz SCS.^5^ Another similar study in 2019 found that 42% of VS patients were sensitive to 60 Hz SCS.^6^ A recent study on 110 patients with DOC who underwent 70 Hz SCS showed that 35 patients were treated effectively.^7^ The fluctuation in the effective rate between study groups suggested the lack of understanding of the SCS mechanism for the recovery of consciousness, highlighting the need for further in‐depth studies.

Currently, 60–100 Hz SCS is commonly used to accelerate the recovery of consciousness in patients with DOC.^5^, ^6^, ^7^, ^8^ Yamamoto et al. also achieved promising results using a lower frequency of 5 Hz SCS.^9^ On this basis, our research group further conducted a cohort study using 5 and 70 Hz short‐term SCS (stSCS) for treating DOC. We confirmed that stSCS effectively increased the level of consciousness, but different individuals had different SCS frequency dependence.^10^ This might be because the brain is a loose collection of multiple oscillators nested at different frequencies. Among these, various neural networks have their corresponding intrinsic frequencies. The resonance triggered by artificially applied electrical stimulation of the same frequency leads to signal amplification and activation of that neural loop to perform a specific neural function.^11^ Therefore, the correct selection of the individualized treatment frequency of SCS is crucial.

Coma recovery scale‐revised (CRS‐R) is commonly used to assess the level of consciousness in patients with DOC. However, most current studies have found that a single session of noninvasive electromagnetic stimulation doesn't cause significant changes in CRS‐R.^12^, ^13^, ^14^ Therefore, the inability of patients with DOC to feed back stimulation effects poses a great difficulty in selecting parameters, which is different from the pain treatment where SCS parameter are adjusted according to subjective patient reports. Electroencephalography (EEG) is particularly suitable for assessing the occult conscious activity of immobile patients with DOC due to its bedside continuous monitoring feature. The debiased weighted phase delay index (dwPLI) measures the actual information interaction between brain regions by excluding the zero‐phase lag generated by the mixture of real and fictitious relations.^15^ Many studies demonstrated that the functional connectivity measured by dwPLI and its brain network topological features based on the graph theory sensitively reflected the changes in the level of consciousness and cognitive function in anesthesia, sleep, DOC and cognitive impairment.^16^, ^17^, ^18^, ^19^, ^20^ A cross‐over sham‐controlled study reported that patients with DOC didn't outwardly display any behavioral improvement after a single stimulation session of transcranial direct current stimulation (tDCS), but the EEG suggested a significant increase of theta band dwPLI in the frontal.^21^ This demonstrated the ability of connectivity indicators characterized by dwPLI to sensitively quantify instant brain effects and provide an immediate basis for parameter adjustment.

Some previous studies by our team found that 70 Hz SCS has a more specific modulatory effect on brain function compared to other frequencies, especially enhancing long‐range functional connectivity.^22^, ^23^ And subsequent clinical study of stSCS for DOC also found its unique delayed effect.^10^ Based on the aforementioned evidence, we proposed a test treatment using a 2‐week puncture stSCS, which had a minimally invasive advantage and a short postoperative recovery period before the patients underwent permanent SCS operation. At the same time, the study combined behavioral and EEG evidence to evaluate the effectiveness of postoperative EEG test for 70 Hz treatment frequency selection and screened its feedback biomarkers for rapid quantification of the modulatory effects based on dwPLI and its brain network properties. Meanwhile, the study also aimed to investigate the brain network response mechanism induced by SCS with the recovery of consciousness going.

## 2 MATERIALS AND METHODS

### 2.1 Study participants

This study recruited 15 MCS and 8 VS patients from December 2021 to August 2022 at Beijing Tiantan Hospital.

All enrolled patients met the following inclusion criteria: (1) diagnosis as DOC; (2) age 14–70 years regardless of sex; (3) duration of disease more than 1 month; (4) a stable stage of consciousness and ineffective conventional treatment for the recovery of consciousness; and (5) no cranial defects or extensive cranial repair.

The exclusion criteria were as follows: (1) concurrent neurodegenerative diseases and life‐threatening diseases; (2) expected survival time less than 3 months; (4) duration of disease more than 12 months; (5) epilepsy not controlled using drugs; (6) spinal fracture and significant spinal stenosis.

This study was designed and conducted in accordance with the Declaration of Helsinki established by the World Medical Association. It was approved by the ethics committee of Beijing Tiantan Hospital in November 2021 (No. KYSQ 2021‐396‐01). And, the trial was registered on the Chinese Clinical Trial Register (ChiCTR2200061278). The patients' immediate family members signed the written informed consent.

### 2.2 Surgical procedure

**FIGURE 1:**
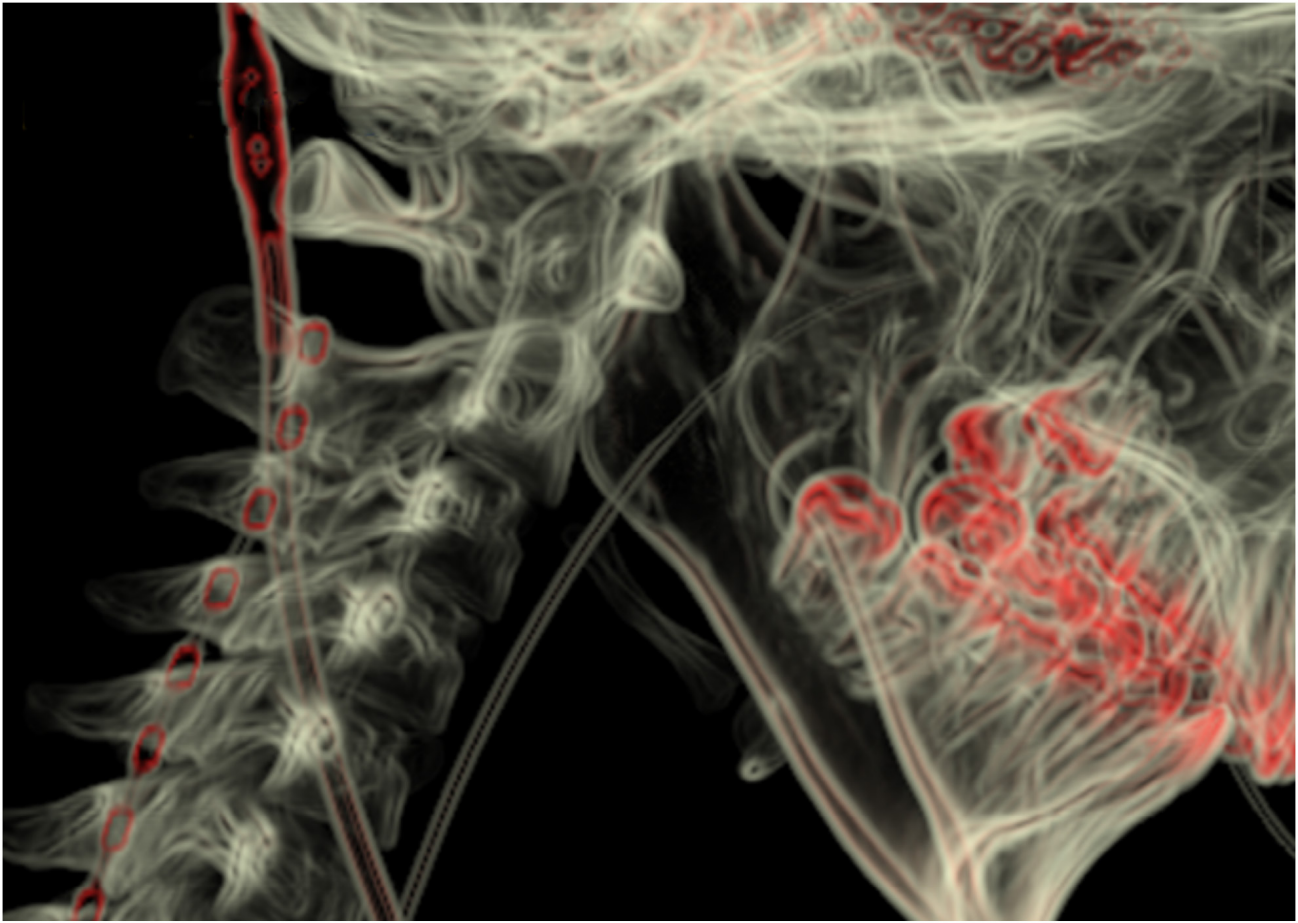
3D reconstruction of cervical CT shows electrode position at epidural space of the C2 vertebrae after short‐term single spinal cord stimulation implantation.

The patients were placed in the prone position after general anesthesia. The skin was punctured at the T7/8 level, and a single row of eight‐contact stimulating electrodes (3777; Medtronic) was delivered upward along the midline of the spinal cord into the epidural space at the C2 level under X‐ray. The electrode extension was connected with the external pulse generator and battery (37022; Medtronic). The cervical spine CT was reviewed within 24 h after the operation to rule out spinal cord injury, epidural hematoma, and electrode displacement (Figure 1). The electrodes were removed at the bedside after 2 weeks of continuous stimulation.

### 2.3 Stimulation protocol

**FIGURE 2:**
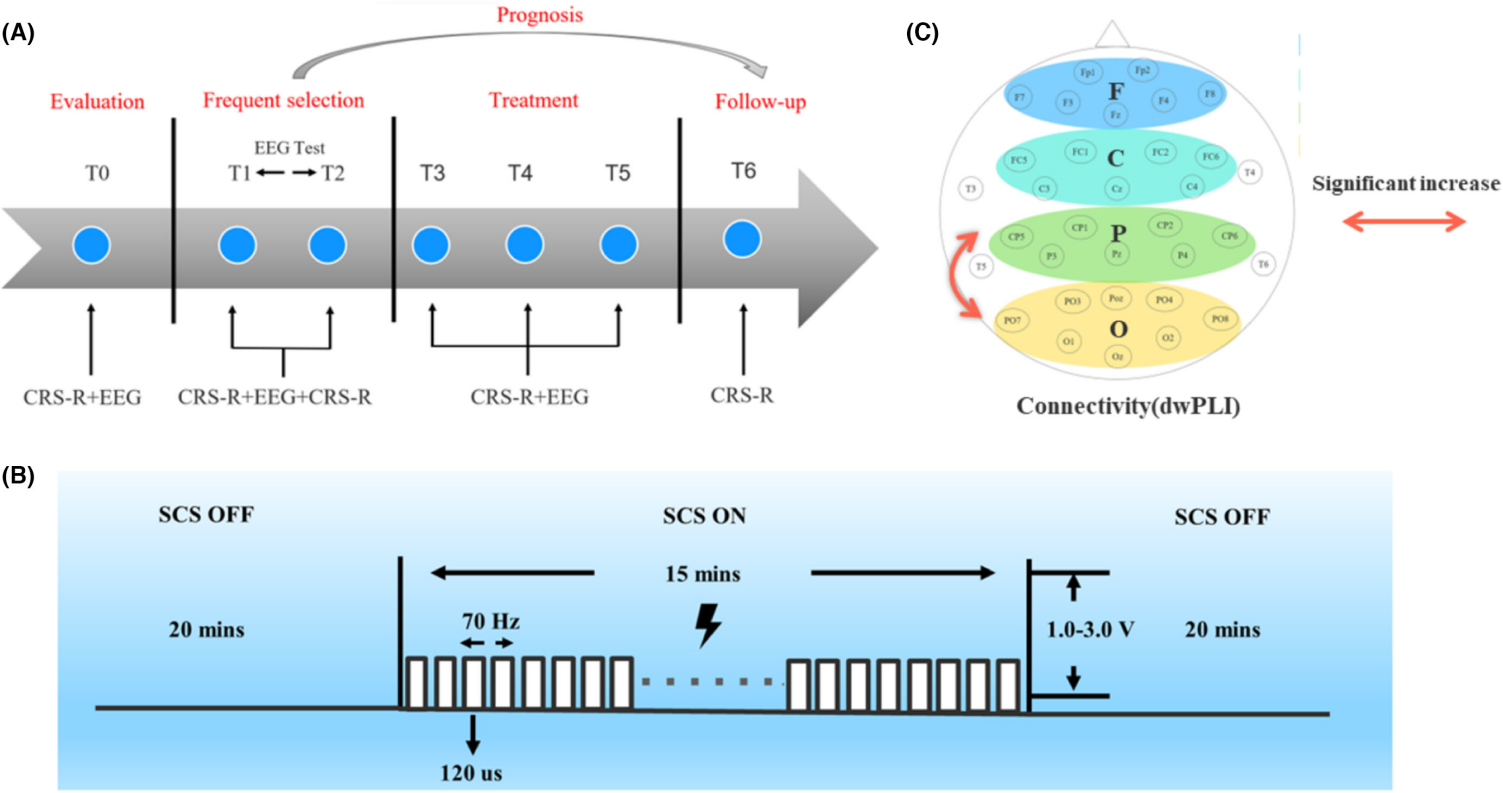
(A) Study flow diagram. T0 preoperative assessment; T1 before single SCS session; T2 after single SCS session; T3 1 week of treatment; T4 2 weeks of treatment;T5 1 week after the treatment; T6 3‐month follow‐up. ns, not statistically significant. Asterisk indicates significant differences (*p < 0.05; **p < 0.01). (B) The stimulation paradigm of electroencephalography test; (C) brain regions of interest contain four brain regions (F, frontal area; P, parietal area; O, occipital area) and PO connectivity in the effective group showed significant changes in at T5 compared to T0.

The uppermost three‐contact bipolar mode (0–1 + 2+) was used. In addition, the pulse width was set to 120 μs, and the frequency was 70 Hz. The stimulation intensity was set to a submotor threshold without significant painful expression. After determining the parameters, EEG tests within 2 days after the operation were performed, including a single session of 15‐min continuous SCS performed under EEG monitoring, and 20‐min online EEG recordings obtained before (T1) and after (T2) stimulation (Figure 2B). Then, two experienced electrophysiologists were offline and independently observed changes in visual EEG background activity before and after stimulation. An increase in alpha rhythm (8–13 Hz) or a decrease in delta rhythm (1–4 Hz) was considered as a good EEG reactivity for 70 Hz stimulation.^10^ The patients who can react to 70 Hz were included into study.

Formal treatment lasted for 2 weeks and was performed with a cycle of 5 min on and 15 min off for at least 12 h every day. The stimulation was turned off at 8 p.m. to accommodate the patient's wake–sleep rhythm.

### 2.4 Clinical assessments and follow‐up

In this study, the CRS‐R was used to assess the state of consciousness at different time points in three phases (Figure 2A). The first phase was the preoperative assessment period, in which the CRS‐R was assessed at least three times in 1 week and the highest score was taken as the baseline CRS‐R (T0). The second phase was the treatment period, including 1 week of treatment (T3), 2 weeks of treatment (T4), and 1 week after the end of treatment (T5). The third phase was the follow‐up period conducted 3 months after discharge (T6) and the follow‐up assessment of CRS‐R was obtained through inpatient, outpatient, surrogate assessment by other medical institutions, or telephone follow‐up to observe the distant delayed effect of stSCS.

Finally, treatment effectiveness was defined as a diagnostic improvement in level of consciousness according to follow‐up results.

### 2.5 EEG recording

We used the Nicolet EEG recording amplifier (EEG V32, Natus Neurology) with 32 Ag/AgCl electrodes to collect online 20‐min resting EEG data at six time points (T0–T5) (Figure 2A). The sampling rate was 500 Hz. All electrodes used FCz as the reference electrode and AFz as the ground electrode. The impedance between the electrodes and the patient's skin was always kept below 5 kΩ.

### 2.6 EEG data preprocessing and analysis

#### 2.6.1 Preprocessing

Raw EEG data were preprocessed offline in the EEGLAB software (version 2021.1) of MATLAB (version 2020b). The steps were as follows: (1) manual removal of obvious noise segments and interpolation processing for bad conductors; (2) use of notch filters to remove powerline interference and 1–40 Hz bandpass filtering; (3) down‐sampling to 250 Hz; (4) removal of oculoelectricity and myo electricity by ICA; (5) segmentation of the data with an epoch of 10 s; (6) adoption of absolute threshold method to remove the segment with noise (±150 μV); and (7) use of average reference for re‐reference.

After data preprocessing, we finally retained the clean data including 90 epochs and divided the data into four frequency bands: delta (1–4 Hz), theta (4–8 Hz), alpha (8–13 Hz), and beta (13–30 Hz).

#### 2.6.2 Debiased weighted phase lag index

The dwPLI was used to estimate the spectral connectivity between channel pairs, which had the advantage of removing volume conduction effects and bias caused by sample size.^24^, ^25^

A 32 × 32 adjacency matrix was obtained by measuring dwPLI under 32 channels. We divided the whole brain into four areas of interest to facilitate subsequent connectivity analysis of brain regions (Figure 2C): frontal area (FP1, FP2, F3, Fz, F4, F7, F8), central area (FC1, FC2, FC5, FC6, Cz, C3, C4), parietal area (CP1, CP2, CP5, CP6, Pz, P3, P4), and occipital area (PO3, PO4, PO7, PO8, Oz, O1, O2). We further analyzed the functional connections between brain regions.

#### 2.6.3 Brain network properties

Before calculating the properties of the brain network, we selected different thresholds for the four frequency bands to construct the brain functional network due to connectivity differences of neural oscillation in different frequency bands. At present, we calculated the small world coefficient corresponding to all the connection matrices under different thresholds which ranged from 0.05 to 0.8 and the step size was 0.05^26^ (calculation method was shown in Appendix S1). Then, we used Mann–Whitney *U* test and found that when the thresholds were selected in order as 0.05, 0.05, 0.05, and 0.1, *p*‐value of small world coefficient was the smallest in the four frequency bands between the effective and ineffective groups (detailed data can be seen in Data S1). Then, the dwPLI connection matrix was thresholded based on the absolute weight size. Any weights below a specified threshold, as well as self‐connections on the main diagonal, were set to 0. This resulted in a weighted connectivity matrix, which was then binarized to create a binary connectivity matrix.

The CC in the graph theory reflects the integrity and interconnectivity of a smaller network and the processing of local information. The CC of node *i* is defined as the ratio between the actual number of edges between all neighbor nodes directly connected to node *i* (excluding node *i*) and the maximum possible number of edges between these neighbor nodes, which can be defined as:

$$C_{i}=\frac{2\sum_{j=1}^{n}v_{\mathrm{ij}}}{n_{i}\left(n_{i}-1\right)}$$

where i and j refer to the electrode number; $v_{\mathrm{ij}}$ is 1 if a suprathreshold connection exists between electrodes i and j, and 0 otherwise; and n is the number of electrodes with suprathreshold connections with electrode i.

The average path length (PL) of the network is opposite to the CC, which reflects the overall efficiency of information integration between different brain regions. It is defined as the average of the shortest paths between all pairs of nodes.

$$L=\frac{1}{N\left(N-1\right)}\sum_{i\neq j}l_{\mathrm{ij}}$$

where N represents the number of nodes in the network, and $l_{\mathrm{ij}}$ represents the shortest path length between nodes i and j.

### 2.7 Statistical analysis

For the baseline clinical data, the measurement data were tested by an independent‐sample *t* test between effective and ineffective groups. The 2 × 2 Fisher exact test was used for the count data. For CRS‐R scores, the data were analyzed by a two‐way repeated‐measures analysis of variance (ANOVA). Bonferroni corrections were used for post hoc multiple comparisons.

For EEG data which conformed to normal distribution, the data in T1 and T2 were tested by the independent‐sample *t* test. Subsequently, for each EEG frequency, one‐way repeated measures ANOVA was used to analyze the main effects of time (T0, T3, T4, and T5) for two groups, and post hoc multiple comparisons were performed using the least significant difference test. However, for EEG data which didn't conform to normal distribution, the data in T0, T3, T4, and T5 were tested by Friedman test.

Finally, the differences between EEG and CRS‐R at different time points were calculated, and their correlations were analyzed using Pearman correlation analysis. All the aforementioned statistical analyses were performed on SPSS (version 26, IBM). A *p* value < 0.05 for the two‐sided test indicated a statistically significant difference and Shapiro–Wilk was used for the normality test of all measurement data.

## 3 RESULTS

### 3.1 Clinical outcome of treatment

After EEG testing, 18 DOC patients (12 MCS and 6 VS) were finally included in the study (Appendix S1). The study comprised 12 male and 6 female patients, and the number of traumatic brain injuries and strokes was half and half. Their age was (41.72 ± 19.36) years, the duration of disease was (5.28 ± 2.87) months, and preoperative CRS‐R score was (8.00 ± 2.87). All patients received complete treatment with a mean voltage of (1.82 ± 0.48) V. After 3 months of follow‐up, 10 patients were found to have a good prognosis at follow‐up and classified as a effective group. There were no significant differences in demographic and clinical characteristics between the effective and ineffective groups (Table 1).

**TABLE 1:** Clinical baseline information between effective and ineffective groups.

Variables	Effective Group (*n* = 10)	Ineffective Group (*n* = 8)	Statistic value	*p*‐Value
Gender				
Male	8	4	NA^a^	0.321
Female	2	4		
Age	35.50 ± 18.45	49.50 ± 18.65	1.592^b^	0.131
Etiology				
TBI	6	3	NA^a^	0.637
NTBI	4	5		
Post‐injure (month)	4.10 ± 1.66	6.75 ± 3.45	1.993^b^	0.075
CRS‐R (T0)	8.80 ± 3.45	7.00 ± 1.60	1.354^b^	0.194
Diagnosis				
VS	3	3	NA^a^	1
MCS	7	5		

*Note*: Gender (F and M); Etiology (TBI and NTBI); Clinical Diagnosis (VS and MCS); CRS‐R. T0 preoperative assessment.

Abbreviations: CRS‐R, coma recovery scale‐revised; F, female; M, male; MCS, minimally conscious state; NTBI, not traumatic brain injuries; TBI, traumatic brain injuries; VS, vegetative state.

^a^ Fisher exact test.

^b^ Independent‐sample *t* test.

**FIGURE 3:**
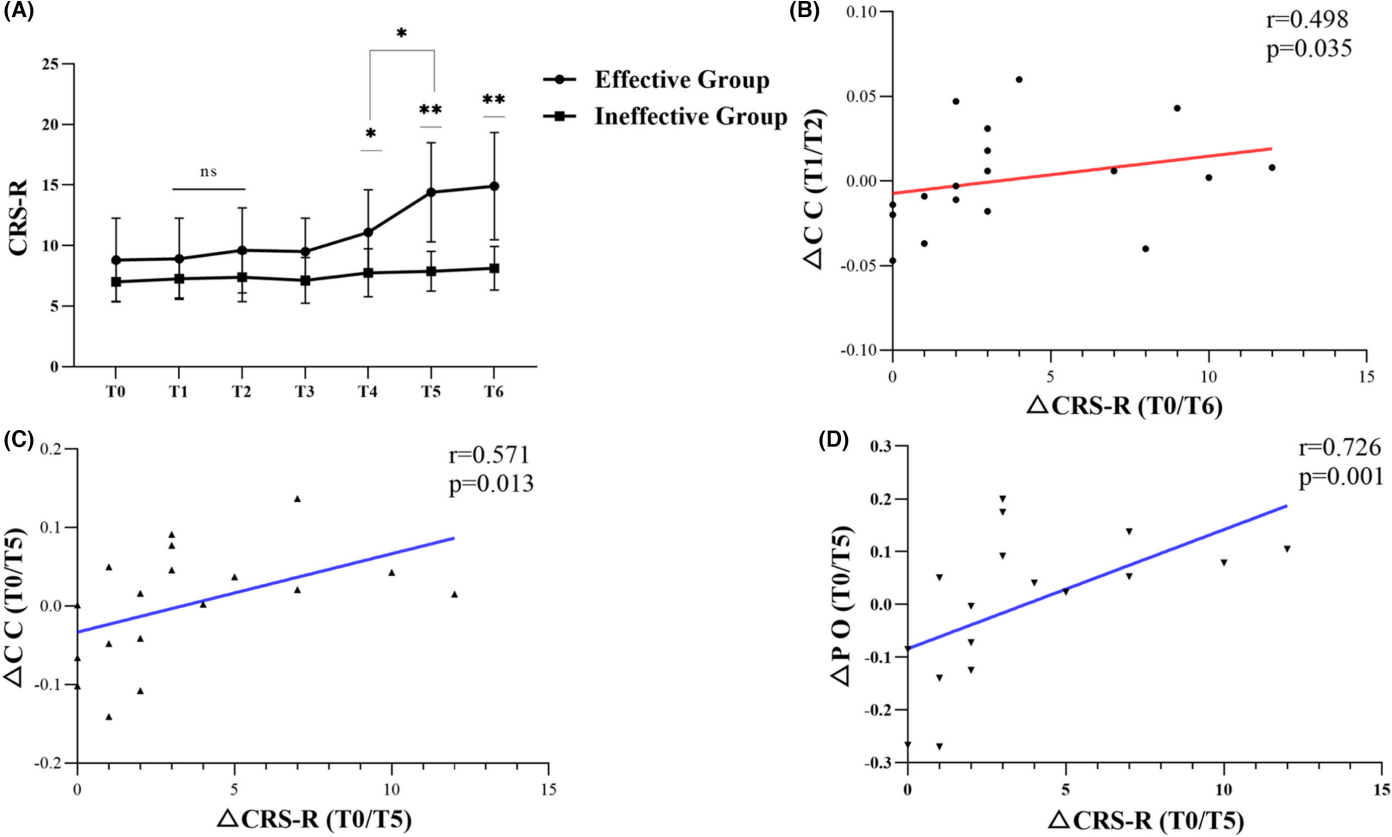
(A) CRS‐R changes at six‐time points in two groups. T0 preoperative assessment; T1 before single SCS session; T2 after single SCS session; T3 1 week of treatment; T4 2 weeks of treatment;T5 1 week after the treatment; T6 3‐month follow‐up. (B–D) Correlation analysis between the respective changes in CRS‐R and beta EEG indexes. The △ indicates the difference between the two‐time points. CC, clustering coefficients; CRS‐R, coma recovery scale‐revised; PO, parietal‐occipital connectivity.

No statistically significant differences were observed in CRS‐R before and after a single SCS session in the two groups (*p* > 0.05; Figure 3A). Further, two‐way repeated measures ANOVA of time (T0, T3, T4, T5, and T6) × grouping (effective and ineffective groups) revealed a significant main effect of both time [*F* (1.739, 27.820) = 20.038, *p* < 0.001] and grouping [*F* (1, 16) = 10.705, *p* = 0.005] factors. Also, a significant interaction effect was observed between the two factors [*F* (1.739, 27.820) = 55.833, *p* = 0.001]. The post hoc analysis in the effective group revealed significantly higher CRS‐R at T4 (*p* = 0.032), T5 (*p* = 0.005), and T6 (*p* = 0.004) than at T0. After the end of treatment, CRS‐R continued to improve in T5 (*p* = 0.014) and T6 (*p* = 0.009) compared with T4 (Figure 3A). However, no statistically significant difference in CRS‐R was found between T5 and T6 (*p* > 0.1). In contrast, the post hoc analysis in the ineffective group showed no statistically significant difference in CRS‐R at each time point (*p* > 0.05).

### 3.2 Effect of single SCS session on EEG

**FIGURE 4:**
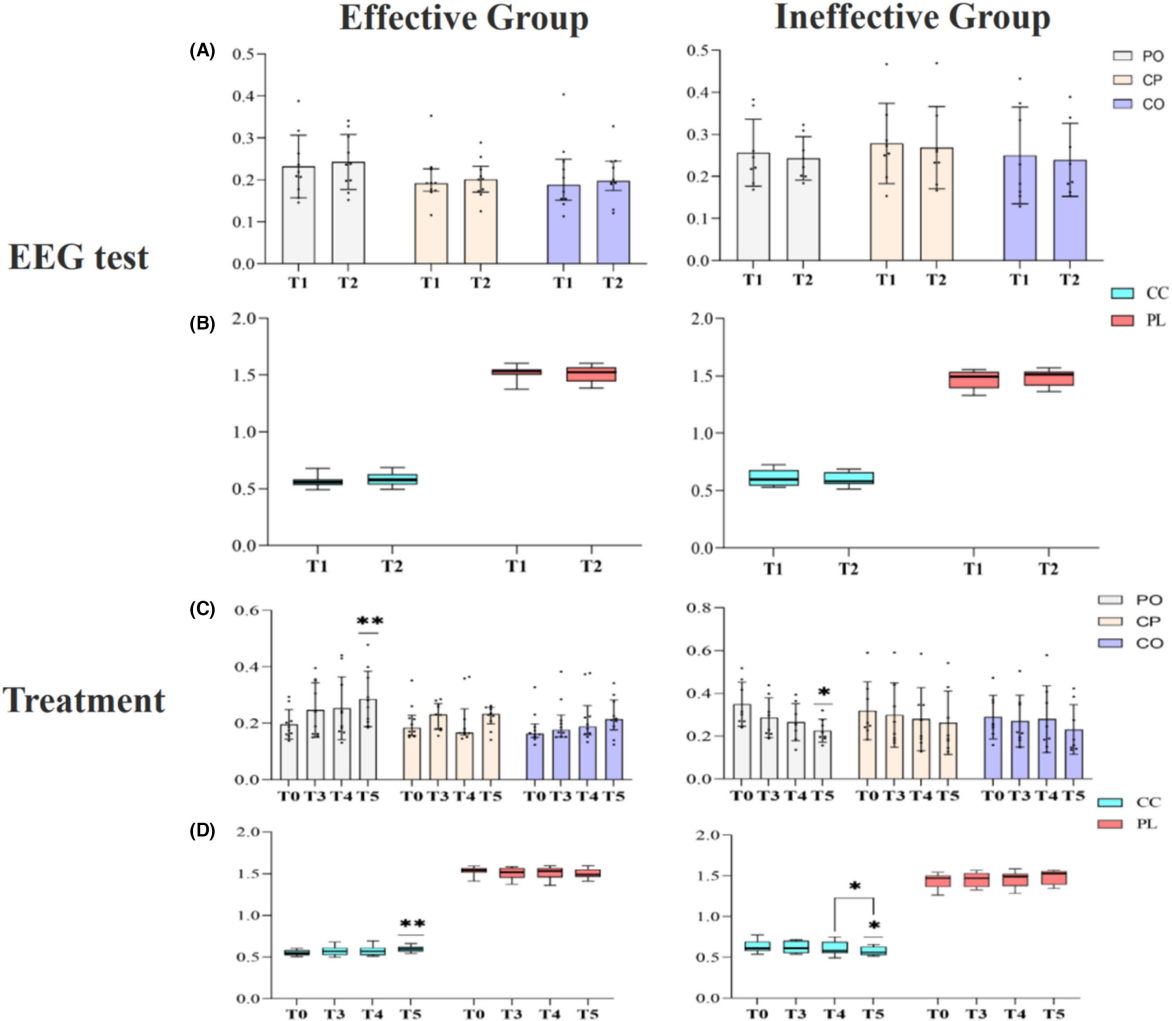
Change of beta EEG indexes of two groups in different time points including EEG test (A) and (B) and treatment (C) and (D). CP, central‐parietal connectivity; PO, parietal‐occipital connectivity; CO, central‐occipital connectivity; CC, clustering coefficient; PL, average path length. T0 preoperative assessment;T1 before single SCS session;T2 after single SCS session;T3 1 week of treatment;T4 2 weeks of treatment;T5 1 week after the treatment. Asterisk indicates significant differences (*p < 0.05; **p < 0.01).

In the effective group, the central‐occipital (CO), parietal‐occipital (PO), and central‐parietal (CP) connectivities and CC in the beta band showed an increasing trend and a decreasing trend in the PL after a single SCS session, while the ineffective group showed the opposite trend (Figure 4A,B and Table 2). However, no statistically significant difference (*p* > 0.05) was found between the aforementioned indicators before and after a single SCS session in both groups. In addition, no significant trends were found in connectivity between other brain regions (Appendix S1).

**TABLE 2:** Dynamic changes in CRS‐R and EEG indexes in the beta band over time.

	T0	T1	T2	T3	T4	T5
(Mean ± SD)						
Effective group						
CRS‐R	8.800 ± 3.458	8.900 ± 3.348	9.600 ± 3.502	9.500 ± 2.759	11.100 ± 3.510	14.400 ± 4.088
PO	0.196 ± 0.521	0.232 ± 0.741	0.242 ± 0.658	0.247 ± 0.964	0.253 ± 0.111	0.286 ± 0.099
CP^a^	0.184 (0.157–0.228)	0.192 (0.173–0.226)	0.201 (0.171–0.232)	0.231 (0.178–0.269)	0.167 (0.158–0.251)	0.233 (0.198–0.256)
CO^a^	0.163 (0.150–0.198)	0.188 (0.152–0.250)	0.198 (0.175–0.244)	0.177 (0.152–0.229)	0.188 (0.160–0.262)	0.214 (0.177–0.283)
CC	0.546 ± 0.032	0.563 ± 0.050	0.576 ± 0.058	0.572 ± 0.061	0.574 ± 0.061	0.595 ± 0.036
PL	1.531 ± 0.050	1.520 ± 0.060	1.510 ± 0.070	1.504 ± 0.075	1.507 ± 0.077	1.495 ± 0.056
Ineffective group						
CRS‐R	7.000 ± 1.604	7.250 ± 1.581	7.375 ± 1.996	7.12 ± 1.880	7.75 ± 1.980	7.88 ± 1.640
PO	0.349 ± 0.103	0.256 ± 0.080	0.243 ± 0.051	0.225 ± 0.053	0.266 ± 0.086	0.225 ± 0.053
CP	0.319 ± 0.135	0.279 ± 0.095	0.269 ± 0.098	0.299 ± 0.150	0.280 ± 0.147	0.262 ± 0.148
CO	0.289 ± 0.102	0.250 ± 0.114	0.239 ± 0.087	0.270 ± 0.121	0.280 ± 0.156	0.231 ± 0.115
CC	0.632 ± 0.775	0.610 ± 0.074	0.596 ± 0.060	0.622 ± 0.070	0.609 ± 0.087	0.576 ± 0.054
PL	1.436 ± 0.094	1.468 ± 0.082	1.487 ± 0.073	1.453 ± 0.088	1.460 ± 0.101	1.487 ± 0.089

*Note*: EEG (CP; PO; CO; CC; PL); time point (T0 preoperative assessment; T1 before single SCS session; T2 after single SCS session; T3 1 week of treatment; T4 2 weeks of treatment; T5 1 week after the treatment).

Abbreviations: CC, clustering coefficient; CO, central‐occipital connectivity; CP, central‐parietal connectivity; CRS‐R, coma recovery scale‐revised; EEG, electroencephalography; PL, average path length; PO, parietal‐occipital connectivity.

^a^ Indicates data didn't conform to a normal distribution and was described by the median (interquartile‐range P25–P75).

### 3.3 Effect of stSCS on EEG during treatment

In the effective group, one‐way repeated‐measures ANOVA (T0, T3, T4, and T5) revealed a time main effect of PO connectivity [*F* (3, 27) = 4.391, *p* = 0.012] and CC [*F* (3, 27) = 3.405, *p* = 0.032] in the beta band. The post hoc analysis showed significantly higher PO connectivity (*p* = 0.002) and CC (*p* = 0.005) in the beta band at T5 compared with T0 (Figure 2C). In terms of the trend over time, the connectivity of CP, PO, and CO (Figure 4C) and CC in the beta band showed an increasing trend, whereas PL showed a decreasing trend (Figure 4D) during treatment. The beta PO connectivity (T3: 0.247 ± 0.096 vs. T4: 0.253 ± 0.111; *p* = 0.769) and CC (T3: 0.572 ± 0.061 vs. T4: 0.574 ± 0.061; *p* = 0.820) at T4 almost growth stalled relative to T3.

The connectivity of CP, PO, and CO and brain network properties of CC and PL in the beta band also showed completely opposite trends during the treatment in the ineffective group compared with the effective group. A time main effect of PO connectivity [*F* (3, 21) = 4.684, *p* = 0.012] in the beta band and the CC [*F* (3, 21) = 3.823, *p* = 0.025] was observed. The post hoc analysis revealed that the beta PO connectivity (*p* = 0.013) decreased significantly at T5 compared with T0 (Figure 4C). The beta CC was significantly lower at T5 compared with T0 and T4 (*p* < 0.05; Figure 4D). Likewise, time main effects of connectivity between other brain regions were also not found (Appendix S1).

### 3.4 Correlation analysis of changes in EEG and CRS‐R

We performed a correlation analysis between behavioral scores and EEG characteristics before and after a single SCS session and before and after treatment. The results showed no significant correlation (*p* > 0.05) between the changes in EEG and CRS‐R at T1/T2 in all patients, but the changes in beta CC at T1/T2 were significantly positively correlated with the changes in CRS‐R at T0/T6 (*r* = 0.498, *p* = 0.035; Figure 3B). We also found that the changes in PO connectivity (*r* = 0.726, *p* = 0.001) and CC (*r* = 0.571, *p* = 0.013) of the beta band at T5/T0 were also significantly positively correlated with the changes in CRS‐R at T5/T0 (Figure 3C,D).

## 4 DISCUSSION

SCS has received increasing attention in DOC treatment, but the difficulties in adjusting stimulation parameters have greatly limited its clinical effect. Many interesting attempts have been made to explore the changes in the brain effects of DOC with different frequencies of SCS.^22^, ^23^ However, they mostly focused on the immediate effects without corresponding clinical behavioral changes to support them. The results in this study showed that the beta CC during the EEG test was significantly correlated with a good prognosis at a 3‐month follow‐up. Furthermore, the beta PO connectivity and CC tended to increase with 2 weeks of treatment in the effective group, but the ineffective group displayed a decreasing trend. This indicated that inappropriate frequency selection could even bring about the significant depression of brain activity and limit the recovery of consciousness in the long term. Therefore, individualized frequency selection based on EEG is particularly important.

The duration of stimulation is also an essential factor influencing the effect of treatment. Patients with DOC often needed to receive electromagnetic stimulation for 10 consecutive cycles and more to induce behavioral changes, probably because the severely impaired consciousness network remodeling in DOC depends on the long‐term potentiation caused by repeated stimulation.^27^, ^28^ Similar results were obtained in this study, where we found a significant increase in CRS‐R at T4. However, a significant delayed effect of 70 Hz SCS was observed, and the CRS‐R continued to increase at T5 in this study. Similarly, beta PO connectivity and CC also suggested a significant increase at T5 than T0, but a stagnant growth was found between T3 and T4.

The power of the beta/gamma band in the posterior cingulate gyrus (PCC) and parahippocampal gyrus was found to be significantly lower under 40 Hz stimulation for 1 week compared with baseline in a study of SCS for pain.^29^, ^30^ Another magnetic resonance imaging study found that 22 patients having pain treated with SCS for 3 months showed significant reductions in the volume of the inferior frontal gyrus, precuneus, posterior cerebellar lobe, and middle temporal gyrus.^31^ The underlying mechanism might be that the marked inhibition of neural activity in the posterior and central brain regions induced by SCS was due to persistent stimulation blocking the normal afferent input of sensory signals from the spinothalamic tract. Higher frequency and lower voltage were developed, such as high‐dose SCS (HD‐SCS) and high‐frequency SCS (HF‐SCS), to overcome the fact that conventional SCS with high voltage induce abnormal sensation.^32^ Numerous studies confirmed that these two stimulation paradigms had significant activation effects in the supratentorial brain regions.^30^, ^31^, ^33^, ^34^ For example, the connectivity between FC3 and TP9 in the beta band was significantly enhanced with HD‐SCS stimulation.^33^ Buentjen et al. also reported HF‐SCS increased power in beta/low‐gamma band compared with baseline.^35^ Thus, evidence suggests that SCS with higher frequency and lower voltage may effectively facilitate signal upload. Our study group previously treated patients with DOC using a single SCS session with voltage 3.0 V and frequency 70 Hz and found an increase in PL, a decrease in CC, and a decrease in small‐world properties during stimulation, suggesting that the overall network converged to a random network. In addition, the connectivity in the gamma band within the frontal lobe still appeared to decrease after stimulation.^25^, ^34^ In contrast, the beta brain network properties in the effective group of patients with DOC in this study showed the opposite change after a single SCS session, which might be due to the lower voltage (1.82 V) effectively strengthening the thalamocortical connectivity. However, suppression of supratentorial brain activity occurred at T3 with the superimposed stimulation effect. After the stimulation was removed at T4, secondary remodeling of the suppressed brain network caused a significant improvement in consciousness.

The origin of consciousness has been an unanswered question. The current information integration theory proposed that the posterior cortical “hot zone” was sufficient for the experience of consciousness.^36^ One study suggested that the loss of consciousness was accompanied by a breakdown of effective connections between the pallidum and PCC, independent of frontal cortex connections.^37^ In addition, a more pronounced decrease in connectivity between the occipital lobe and other brain regions, as well as a loss of interhemispheric connectivity between angular gyrus and precuneus, was observed in patients with DOC caused by widespread cerebral ischemia and hypoxia.^38^ In terms of brain network topology, the brain network in the human cortex consisted of central nodes in the parietal‐occipital lobe of the posterior brain region and peripheral nodes in the frontal lobe of the anterior brain region.^39^ Central anterogradeization of brain networks under anesthesia has been reported to be a characteristic change in propofol‐induced unconsciousness.^40^ A study further analyzed the dynamics of EEG transients and found that, compared with healthy participants, patients with DOC showed a collapse of the posterior state characterized by high connectivity within the parietal lobe transfer process to other states and an increase in the spontaneous transfer of anterior states characterized by high connectivity within the frontal lobe instead. Spatially, the overall brain network dynamics and extensive disconnections between brain regions reduced in patients with DOC and information was trapped in the frontal lobe, leading to ineffective integration of information.^41^ In conclusion, unconsciousness is closely associated with interrupted functional connectivity and disruption of brain network topology in posterior brain regions.

Recent studies have reported that the beta/gamma neural oscillations in the posterior cortex can be used as a biomarker of consciousness.^42^ Indeed, the results of this study corroborated this hypothesis. We found that increased consciousness after stSCS treatment was accompanied by increased CO, CP, and PO connectivity in the beta band. However, a series of previous studies on the immediate brain effects evoked by a single 70 Hz SCS emphasized the importance of the frontal lobe.^34^, ^36^, ^37^, ^38^, ^39^, ^40^, ^41^, ^42^, ^43^ The difference in brain area activation might be due to the spatial distribution of neural activity in different frequency bands. One study reported significant differences between MCS and VS mainly manifested in the beta activity of the central region.^44^ In addition, the difference also could be attributed to the treatment duration of SCS. The results reported in a prior case also suggested that the treatment of stSCS for 21 days increased the level of consciousness mainly by enhancing the complexity in the parietal lobe.^45^

In conclusion, the effect of 2‐week persistent stimulation in this study was more likely to enhance the information interactions of the local functional module in the brain network. Especially, it increased the beta neuronal synchronous activity in the parietal‐occipital lobe to reproduce the central location of information clustering in the posterior cortical “hot zone,” ultimately facilitating the recovery of the consciousness network. Unfortunately, this study did not find significant changes in low‐frequency neural activity during the treatment, which might be related to the target area of the stimulation intervention. Hermann found that the theta–alpha PO connectivity was a reliable marker of improved consciousness in tDCS responders targeting the DLPFC.^46^ A similar study also found that the changes in left frontal‐parieto‐occipital theta connectivity were positively correlated with the changes in CRS‐R scores after high‐definition tDCS (HD‐tDCS) intervention.^47^ In contrast, a study used HD‐tDCS targeting the precuneus as a treatment for patients with DOC and revealed that improved consciousness was accompanied by an increase in mean PO connectivity in the beta band and the whole brain in the gamma band.^48^ Thus, it was clear that the stimulation on the forehead indirectly activated the posterior brain regions via low‐frequency frontoparietal long‐range connectivity. However, stimulation that directly modulated the posterior brain regions mainly induced high‐frequency neural activity in local regions to facilitate information interactions.

The limitation of this study was the preliminary exploration that the reduced test efficacy due to small sample size resulted in no statistically significant difference in some of the trends. Second, this study only screened the biological indicator of 70 Hz as a treatment frequency. Research on more frequencies and more efficient EEG testing paradigms is warranted. Finally, this study focused on the intervention mechanism of SCS on brain activity in patients with DOC. Hence, the combined spinal–cortical electrophysiological changes under different parameter combinations of frequency and voltage should be explored in the future to comprehensively explain the bottom‐up mechanism of SCS for the recovery of consciousness.

## 5 CONCLUSIONS

We, for the first time, combined the real‐time EEG changes before and after single SCS with longitudinal follow‐up outcomes to explore the immediate biological indicators for individualized frequency selection. The results showed that beta CC using an EEG test may be an immediate feedback indicator for selecting 70 Hz as the treatment frequency for SCS. The study also revealed that SCS might facilitate the recovery of consciousness by enhancing local information interaction in the posterior brain regions, which lay a solid foundation for a stable improvement of consciousness treated by SCS and development of SCS closed‐loop systems in DOC patients.

## AUTHOR CONTRIBUTIONS

**Jianghong He:** Conceptualization, supervision, funding acquisition. **Yutong Zhuang:** Methodology, data collection and original draft writing. **Qianqian Ge:** Formal analysis and Visualization. **Qinghua Li:** Resources and writing‐editing. **Long Xu:** Validation. **Xiaoli Geng:** Follow up data collection. **Ruoqing Wang:** Data curation.

## FUNDING INFORMATION

This work was supported by the National Natural Science Foundation of China (82272118) and Beijing Medical Award Foundation of China (YXJL‐2022‐09000277).

## CONFLICT OF INTEREST STATEMENT

All authors reported no conflict of interest.

## Supporting information


Data S1:

Appendix S1:

## Data Availability

The raw EEG and behavioral data in the study will be available from the corresponding author.
