# Supplementary material for: Combined behavioral and EEG evidence for the 70 Hz frequency selection of short‐term spinal cord stimulation in disorders of consciousness
Source: CNS Neurosci Ther. 2023 Aug 11;30(2):e14388. doi: 10.1111/cns.14388 (PMC10848050; doi:10.1111/cns.14388)
Supplement: Supplementary file 2 — Appendix S1: [file CNS-30-e14388-s001.docx]

## 一、Demographic details and Changes in CRS-R for patients

| **Patient** | **Gender** | **Age** | **Etiology** | **Post-injure**  **(months)** | **CRS-R**  **(T0)** | **CRS-R**  **(T6)** | **Changes of**  **diagnosis** |
| --- | --- | --- | --- | --- | --- | --- | --- |
| **Effective** |  |  |  |  |  |  |  |
| 1 MCS- | M | 21 | T | 5 | 8 | 18 | Improved to EMCS |
| 2 MCS- | M | 52 | S | 4 | 9 | 16 | Improved to EMCS |
| 3 VS | M | 32 | S | 4 | 7 | 11 | Improved to MCS+ |
| 4 MCS- | M | 64 | S | 8 | 8 | 11 | Improved to MCS+ |
| 5 MCS- | M | 18 | T | 3 | 8 | 20 | Improved to EMCS |
| 6 MCS- | M | 61 | T | 5 | 11 | 14 | Improved to MCS+ |
| 7 VS | M | 34 | T | 2 | 3 | 12 | Improved to MCS- |
| 8 MCS- | F | 14 | T | 4 | 13 | 15 | Improved to MCS+ |
| 9 MCS+ | F | 41 | S | 3 | 15 | 23 | Improved to EMCS |
| 10 VS | M | 18 | T | 3 | 6 | 9 | Improved to MCS- |
| **Ineffective** |  |  |  |  |  |  |  |
| 1 VS | M | 67 | T | 5 | 6 | 6 | Remained VS |
| 2 MCS- | F | 49 | S | 9 | 8 | 8 | Remained MCS- |
| 3 VS | F | 52 | S | 11 | 6 | 7 | Remained VS |
| 4 MCS- | F | 61 | S | 5 | 7 | 9 | Remained MCS- |
| 5 MCS- | M | 30 | S | 5 | 6 | 8 | Remained MCS- |
| 6 MCS- | F | 67 | S | 11 | 10 | 10 | Remained MCS- |
| 7 MCS- | M | 56 | T | 1 | 8 | 11 | Remained MCS- |
| 8 VS | M | 14 | T | 7 | 5 | 6 | Remained VS |

Gender (F female; M male); Etiology (T traumatic brain injury;S Stroke); CRS-R, Coma recovery scale-revised; Clinical Diagnosis(VS Vegetative state; MCS- Minimally Conscious State minus; MCS+ Minimally Conscious State plus; EMCS Emerged from MCS).

## 二、Small-World Coefficient

Now a large number of studies have shown that the brain is neither a complete random network nor a completely ordered network, but has a small-world network property. The so-called small-world network means that it has a relatively small characteristic path length and a relatively large clustering coefficient. In other words, the characteristic path length and clustering coefficient of the small-world network are between the ordered network and the random network. The small world coefficient is defined as:

$$\sigma=\frac{\frac{C_{real}}{C_{random}}}{\frac{L_{real}}{L_{random}}}$$

Where $C_{real}$ and $L_{real}$ are the clustering coefficient and characteristic path length of the network to be analyzed, and $C_{random}$ and $L_{random}$ are the clustering coefficient and characteristic path length of the random network. If the small-world coefficient $\sigma>1$, it means that the network we are studying has the characteristics of a small-world network, otherwise it does not have the characteristics of a small-world network.

## 三、Changes in beta connectivity of EEG at different time-points

|  | **T0** | **T1** | **T2** | **T3** | **T4** | **T5** |
| --- | --- | --- | --- | --- | --- | --- |
| **（Mean ± SD）** | | | | | | |
| **Effective Group** | | | | | | |
| FC^*^ | 0.186  (0.146-0.221) | 0.190  (0.172-0.234) | 0.187  (0.150-0.311) | 0.195  (0.165-0.273) | 0.177  (0.165-0.231) | 0.179  (0.158-0.246) |
| FP | 0.198 ± 0.058 | 0.190 ± 0.055 | 0.197 ± 0.059 | 0.214 ± 0.060 | 0.204 ± 0.070 | 0.208 ± 0.042 |
| FO^*^ | 0.176  (0.158-0.265) | 0.160  (0.153-0.260) | 0.183  (0.150-0.262) | 0.196  (0.164-0.262) | 0.182  (0.166-0.256) | 0.184  (0.159-0.285) |
| **Ineffective Group** | | | | | | |
| FC | 0.288 ± 0.112 | 0.268 ± 0.125 | 0.249 ± 0.120 | 0.283 ± 0.142 | 0.287 ± 0.152 | 0.261 ± 0.145 |
| FP^*^ | 0.234  (0.210-0.361) | 0.221  (0.171-0.300) | 0.209  (0.183-0.291) | 0.244  (0.167-0.312) | 0.217  (0.165-0.340) | 0.204  (0.145-0.341) |
| FO | 0.304 ± 0.121 | 0.263 ± 0.138 | 0.232 ± 0.093 | 0.279 ± 0.107 | 0.291 ± 0.148 | 0.243 ± 0.157 |

EEG（FC frontal-central connectivity; FP frontal-parietal connectivity; FO frontal-occipital connectivity）；Time point（T0 preoperative assessment;T1 before single SCS session;T2 after single SCS session;T3 one week of treatment ;T4 two weeks of treatment;T5 one week after the treatment）. Asterisk indicates data does not conform to a normal distribution and is described by the median (Interquartile-range P25 – P75).
